# A novel laypeople mass-casualty assessment algorithm: LACA

**DOI:** 10.1186/s12245-026-01285-z

**Published:** 2026-07-01

**Authors:** Jan Carlo Del Tedesco, Lion Sieg, Axel R. Heller, Hendrik Eismann

**Affiliations:** 1 Department of Anaesthesiology and Intensive Care Medicine, Hannover Medical School, Carl-Neuberg-Strasse 1, 30625 Hannover, Germany; 2 Department of Anaesthesiology and Surgical Intensive Care Medicine, University Hospital Augsburg, Stenglinstr. 2, 86156 Augsburg, Germany

**Keywords:** Mass casualty incident; Disaster medicine, Triage, PRIOR, mSTART, START, Emergency medicine, Situational awareness; Decision support algorithm

## Abstract

**Background:**

Laypeople, usually the first on scene at a mass casualty event, still have no input into early operational tactics. A new and easy to learn algorithm done by laypeople might gain additional information for operational tactics, especially reinforcement.

**Methods:**

A validated mass casualty data set was used to generate sets of 20 patients. The triage gold standard was compared to severity scores of different already established algorithms as well as the new invented allocation algorithm LACA. For algorithm quality Cronbach’s Kappa, specificity, sensitivity, over- and underassessment was examined. Kullback-Leibler-Divergence was used to show information loss.

**Results:**

The LACA algorithm showed a moderate severity correlation (Cohen’s Kappa = 0.469), lower than mSTaRT or PRIOR. PRIOR showed the already well known and intentional overassessment. LACA showed highest underassessment of all three evaluated algorithms. After adjustment the information loss could be reduced to D_KL_ = 0,0002 bits.

**Conclusions:**

The “LACA” represents a practical and layperson-friendly addition to pre-hospital response strategies in mass casualty incidents. It can provide a reliable initial assessment that supports early operational decision-making. Additionally, this new approach enables emergency dispatch centers to perform a more tailored mobilization of rescue resources in the first place, based on the structured information provided by laypeople or lay responders. To maximize individual survival LACA must be followed by established triage algorithms like mSTaRT or PRIOR applied by trained emergency personnel.

## Introduction

Mass-casualty incidents (MCIs) represent some of the most complex and unexplored entities of emergency medical scenarios. Following recent terroristic events and the growing threats of military conflicts research intensified significantly. To allocate resources of emergency medical services (EMS) and disaster relief units adequately diverse triage algorithms were established. Well-known concepts are the “STaRT” or the modified “mSTaRT” algorithm [1]. In Germany the PRIOR algorithm is a common tool published by the Federal Office of Civil Protection and Disaster Assistance [2]. 

Most triage systems classify patients into three to five categories, combining diagnostic and limited therapeutic steps, e.g. using a tourniquet. Most triage algorithms aim to identify patients who need immediate treatment and transport (so called “find the red”-concept).

However, beyond patient-level prioritization, accurate early situational assessment also influences operational tactics and effectiveness. Dispatch centers heavily dependent on high quality and timely information from the scene to allocate EMS appropriately. Coordinators will likely face a reduction in medical personnel availability in other operational areas. The over- or underestimation can either deplete critical resources or delay vital interventions.

Traditionally, initial evaluation of patients is conducted by the first emergency professionals to arrive at the scene. In Germany, a secondary triage assessment is performed by emergency physicians on-site. In EMS-based systems with a paramedic-only concept, the first medical evaluation by doctors occurs upon arrival at the emergency room.

To date, few studies have investigated the potential role of laypeople, bystanders or non-medical personnel as firefighters or police officers, in providing structures early information during MCIs [3]. Although adequate therapeutical interventions are limited, the primary advantage of integrating non-medical personnel might be the reduction of time delays in reinforcement of emergency response. In this study “laypeople” are defined by “missing professional medical experience”: This includes not only bystanders, but also non-medical response units.

This study introduces the “Laypeople Casualty Assessment Algorithm (LACA)”. It represents a simple, communication-based assessment tool designed to help non-medical individuals to provide dispatch-relevant information in the early stages of MCIs. LACA does not aim to guide clinical decision-making or patient-level prioritization. Instead, it aggregates information about the apparent severity distribution to support early awareness and resource mobilization.

**Fig. 1:**
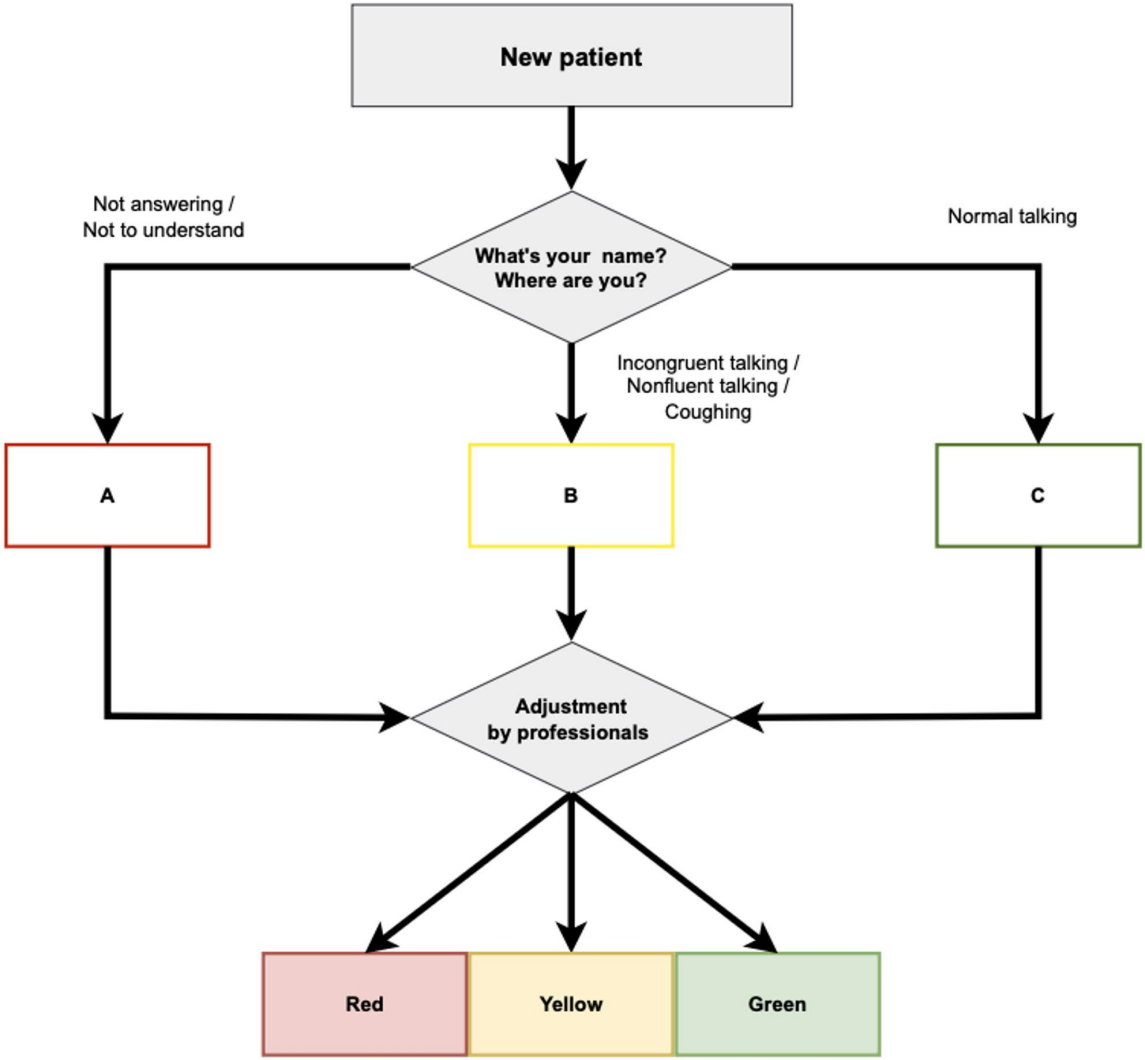
Basic concept of severity assessment via LACA

It could be an accessory tool for motor vehicle drivers’ medical packs. After calling the emergency dispatch and informing about the incident, the layperson is guided to apply a basic triage algorithm if the scene is safe enough. Our non-contact, non-therapeutic algorithm centers on two key questions: “What’s your name?”, “Where are you?”. (Fig. 1) These are generic questions; other questions might fit as well. For categorization, it is not the content that matters, but the manner of response. The potential outcomes after interaction could be:

A: No verbal response or incomprehensible sound; → vital threat.

B: disoriented, incoherent, or coughing → possibly severely injured.

C: Fluent, coherent speech p → ossibly minor injured.

We intentionally label these as A-C to emphasize that they are not equivalent to the categories one to three of medical triage systems.

Unlike pathophysiological or organ system-based approaches, designed for trained rescue personnel - such as the xABCDE or MARCH schemes [4, 5] - our method relies on simple questions. These questions indirectly capture key symptoms and help identify patients in critical condition or those who might require urgent medical intervention. E.g. critical respiratory deficits (as “A and B problems”) might undermine clear answering and can lead to LACA “level A”.

The following analysis explores whether layperson assessments via LACA can approximate the severity distribution of an MCI. A well-established protocol could enable dispatchers to make more informed early decisions regarding resource allocation.

## Materials and methods

### Data and simulation

The simulation is based on a standardized dataset provided by the Federal Office of Civil Protection and Disaster Assistance containing mass casualty cases: Every patient case vignette contains multiple clinical attributes, including vital parameters, pain assessment, injury severity and state of consciousness. Each simulated patient was assessed and categorized through a multi-stage process by a group of experienced emergency physicians [6]. This triage consensus represented the “gold standard”, constituting the baseline for all evaluations. Subsequently, we evaluated each case manually using LACA, PRIOR and mSTART. Index terms derived from the digital patient case vignettes served to define triage categories and severity levels, e.g. the attributes “disoriented” or “not understandable” led to “LACA level B”.

We simulated 30 MCI events, each containing 20 virtual patients – a scale chosen to resemble a typical multi-vehicle collision scenario. There are no cumulative data or register presenting the incidence of mass causality events per year (in Germany). The simulation process was done with Python: First, patients were chosen randomly from the dataset (each case with individual parameters, till 20 cases). Second, case categorization was performed automatically, based on previously established manual mapping.

To analyze dispatch-level patterns rather than individual triage accuracy, we computed the overall distribution of A, B, and C responses per simulated incident and compared these “LACA-derived severity profiles” with the true underlying severity distributions from the reference dataset.

## Statistics

Case sets were analyzed via SPSS (Version 31.0, IBM, 2025) and Excel (Version 16.78.3, Microsoft Cooperation, 2025). Interrater reliability and algorithmic constituency were evaluated using Cohen’s kappa coefficient. Secondly, we estimated the over- and under-triage of PRIOR and mSTART in comparison to the gold standard. Differences in severity distributions between the gold standard and LACA were quantified optimized using highly sensitive Kullback-Leibler-Divergence measurements. It represents a relatively uncommon but useful and extremely sensitive technique for setting cut-off points, discriminating between entities or adjusting tests [7]. As level for significance α < 0.05 was chosen. The graphics were created using GraphPad Prism (Version 10.6, GraphPad Software, 2025).

## Results

The term “triage” was retained for mSTART and PRIOR. LACA does not meet the definition of classical triage algorithms. Medical examination and life-saving interventions are missing. For LACA the terms “overrepresentation” and “underrepresentation” are used.

### Algorithm agreement

Interrater reliability showed significant differences between the gold standard and every tested algorithm (*p* < 0.001). Cohen’s Kappa coefficients ranged between 0.574 (mSTaRT) and 0.469 (LACA), reflecting a moderate interrater agreement across the algorithms assessed [8]. 

**Fig. 2:**
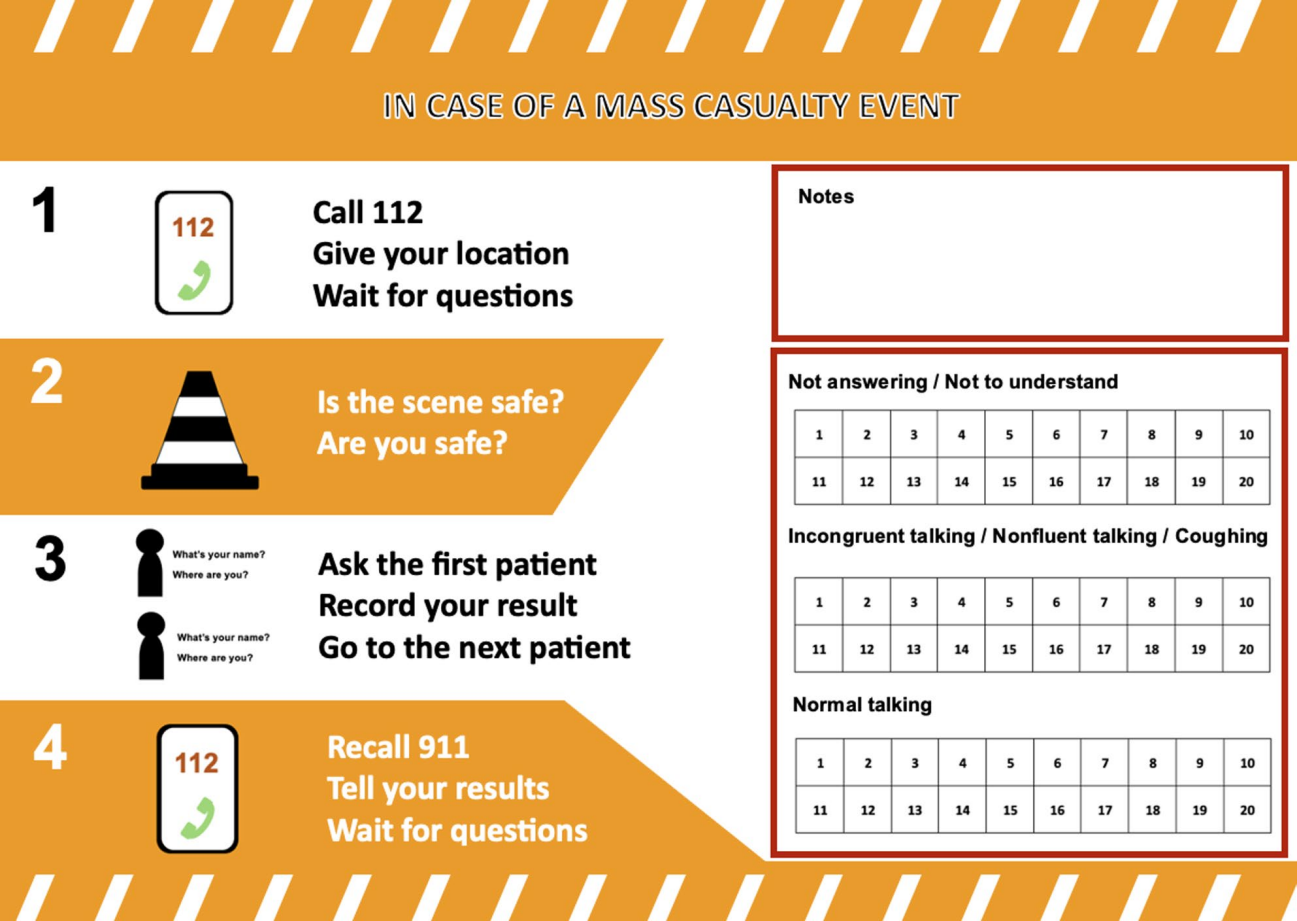
Draft of an information card for laypeople

The cumulative sensitivity for 2000 patients was 1.0 for PRIOR, 0.98 for mSTaRT, and 0.59 for LACA. Cumulative specificity was 0.92 for LACA, 0.92 for mSTaRT and 0.75 for PRIOR (Fig. 2).

### Over- and underassessment of common triage algorithms

The mean for mSTaRT showed, that three patients were overtriaged per 20 people (15.3%, mean = 3.060, SD 1.722) and two patients were undertriaged (9.8%, mean = 1.950, SD 1.290). PRIOR presented increased results with five people overtriaged (23.8%, mean = 4.760, SD 1.902) but only 1 patient undertriaged per set (5.5%, mean = 1.100, SD 1.078).

The new LACA overrepresents the severity of four people on average (18.2%, mean = 3.630, SD 1.773) and underrepresents three (15.1%, mean = 3.010, SD 1.624). Examining the most impactful differences in distribution between the gold standard and LACA reveals that 2 people per 20 patients were misclassified to level II instead of level I (8.8%, mean = 1.76, SD 1.45, cumulative 176 of 2000 patients), whereas three of 20 patients were categorized as level II instead of level III initially (16.9%, mean = 3.38, SD = 1.674, cumulative 338 of 2000 patients).

### Adjustment

To fit level A, B, C of the LACA algorithm with classifications done by mSTaRT, PRIOR or the gold standard adjustment of the output is needed. Fitting the gold standard, LACA would need the following correction factors to recalibrate overall incident severity more reliably.

Level I: level A + (cumulative patients x 8%).

Level II: level B – (cumulative patients x 19%).

Level III: level C + (cumulative patients x 11%).

**Fig. 3:**
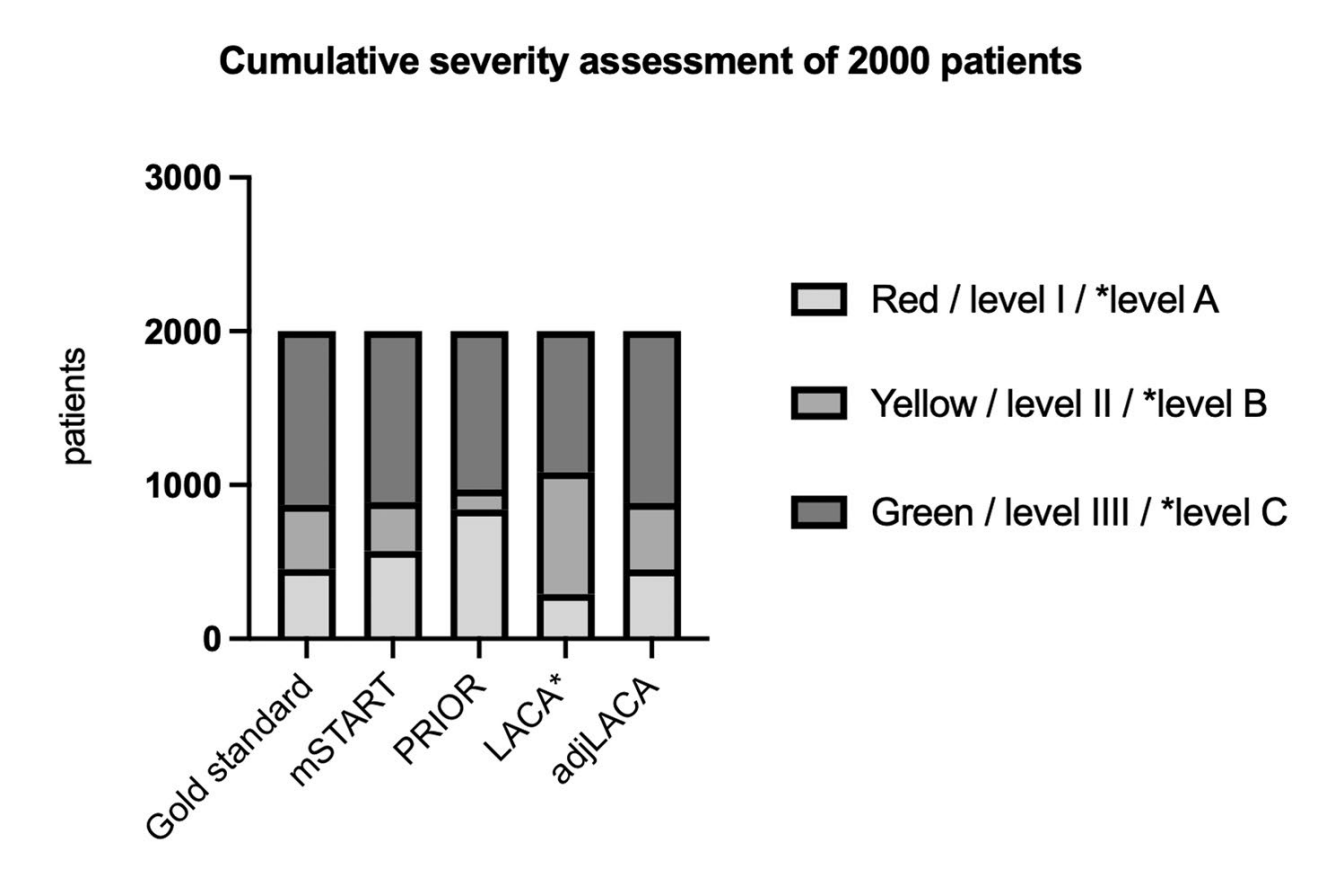
Cumulative triage levels in 2000 patients. Differentiation between different algorithms and approaches. * = formal note to signal that LACA severity assessment are not equivalent to levels of mSTART or PRIOR. AdjLACA = adjusted LACA

Those approximations for an adjusted LACA result are consistent with 20 patients set and the overall evaluation of the cumulative 2000 patients. KL Divergence shows a moderate difference between gold standard and LACA (D_KL_ = 0,1220 bits) (“moderate”), whereas after adjustments adjLACA (adjusted LACA) shows a smaller information loss (D_KL_ = 0,0002 bits) (Fig. 3).

## Discussion

### Sensitivity and specificity of algorithms

The exanimated algorithms demonstrate relevant differences in classification response. As already known and intentional, the PRIOR algorithm tends to over-triage patients to category “I / red / urgent”. It is reflected by its high sensitivity at the cost of reduced specificity. In practice, over-triage would generate a slight overresponse in tactical resource planning. In regions with well-developed civil protection structures, excessive resource allocation may be manageable. However, in areas with limited personnel, this overestimation may become a risk. mSTaRT, in contrast, tends to miss individual critically ill patients, while reducing initial over-triage. LACA demonstrates a notably high capacity to correctly identify non-critical patients, yet fails to detect a substantial proportion of severely injured individuals. If the minimalistic design of LACA should be retained, a mandatory adjustment by trained medical professionals is required. Otherwise, its clinical utility cannot be preserved.

### Conceptual position of LACA

The LACA system is not a clinical triage tool and should not be evaluated as such. There is no adequate examination that allows medical classification or triage, nor basic live-saving actions. Instead, it represents an early information-encoding method designed for non-medical users. It could be seen as a dispatcher-guided reconnaissance. The subsequent adjustment remains central, as it links laypeople assessment to overall MCI severity. Based on our results, medical professionals can reinforce disaster response units adequately using the suggested weighting formula. The algorithms accuracy at the event level – rather than at the individual-patient level – is the key measure of usefulness.

### Focus laypeople

There is a relevant trend involving laypeople in mastering MCI. While small incidents can be addressed via EMS easily, large events involving damaged infrastructure, prolonged resource deficits and mass casualties depend on auxiliary forces. Integrating bystanders with local knowledge, gadgets and simply „helpful hands” seems only consequent and is part of diverse modern relief concepts [9, 10]. While the involvement of laypeople during prolonged incidents is generally accepted, additional individuals are considered undesirable during the so-called “chaos phase.” We propose that a well supervised and guided group of laypeople can support emergency personnel in all phases of disaster response. Besides first aid and securing the emergency location as told in courses for driving licenses, we have to question how laypeople can be integrated in events that are far bigger than a single car crash. In reality, self-initiated and self-organized actions by civilians are a frequently observed phenomenon [11]. Rather than viewing these actions as disruptive, they should be recognized as a potentially valuable resource. Furthermore, we want to empathize that structured and guided helping can significantly reduce trauma and support mental resilience of the bystanders themselves. Otherwise volunteering remains a risk factor to acquire PTSD symptoms [12, 13]. 

**Fig. 4:**
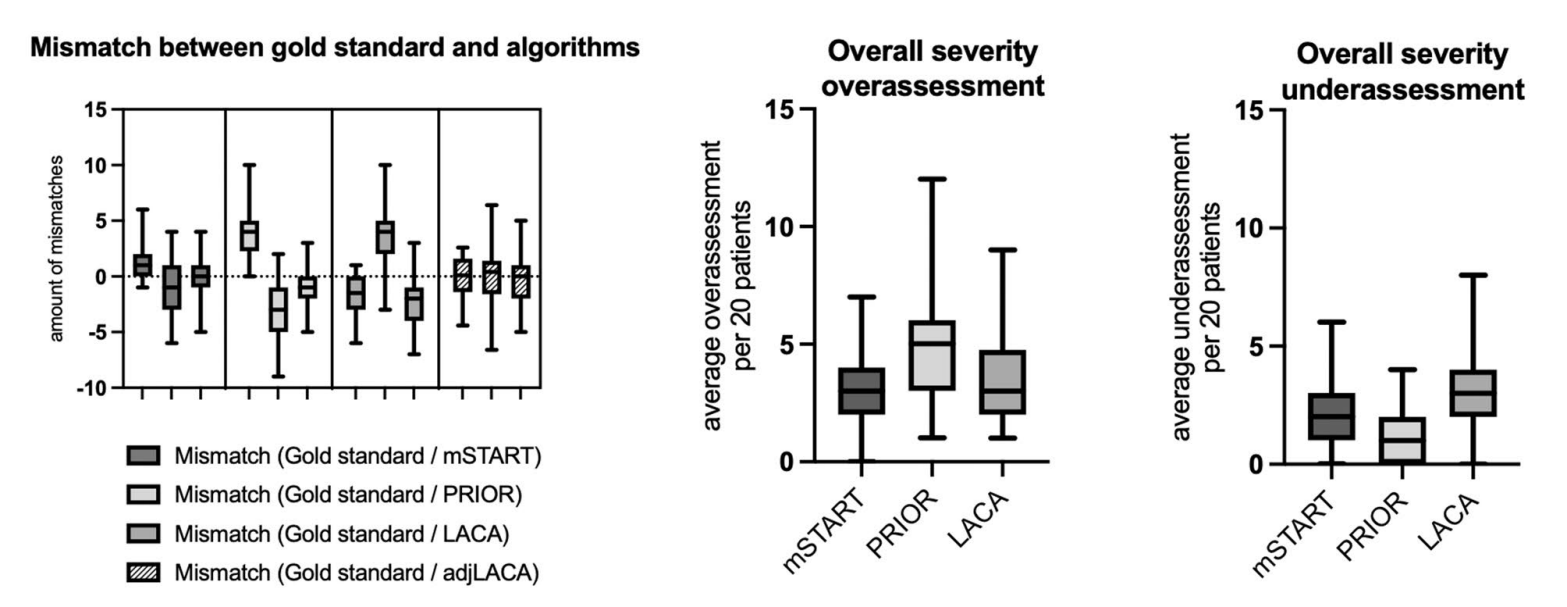
Mismatch, over- and underestimation of severity via different algorithms. Negative numbers represent underestimation, positive numbers represent overestimation. Absolute counts per set can be seen on the right. AdjLACA = adjusted LACA. Mismatch graphs can be interpreted from left to right as category “red”, “yellow”, “green”

Lay severity assessment might be an adequate involvement of bystanders in the first minutes of a disaster. The “LACA card” could be part of first aid kits in cars. (Fig. 4)

### Focus lay responders

LACA demonstrates potential benefit in common car accidents, but also in scenarios characterized by prolonged absence of medical care. Usually, medical units are not allowed to move into hot zones of CBRN disasters or shootings. Lack of first information and therefore time delay can be resolved by so called “lay responders”, emergency personnel without further medical education, e.g., deployed fire fighters or tactical forces. They represent a specialized and highly heterogenous subgroup of (non-medical) laypeople which need further evaluation. Adapting LACA to specific medical knowledge and skills could further enhance its value.

### Limitations and future directions

LACA cannot ensure accurate individual identification of life-threatening conditions and should not replace established triage systems. Further prospective and field-based studies are needed to assess real-world usability by laypeople, time efficiency and integration into command control communication chains. Clarifying these aspects will help determine its operational benefit within disaster management frameworks.

The “LACA card” will undergo further evaluation and optimization. Laypeople might profit from a checklist on the other side, which addresses basic personal safety or potential tasks (e.g. securing the scene correctly, first lifesaving skills).

## Conclusions

The (adj)LACA algorithm can function as a preliminary information gathering tool in MCIs. It enables non-medical bystanders or responders to give structured, approximate severity information to dispatch centers in the critical, early minutes of an MCI. While unsuitable for patient-level prioritization, it can support early reinforcement and resource decisions. Further development and validation are required before operational implementation.

## Data Availability

Supplementary material is available upon reasonable request.

## Declarations

### Use of AI in Manuscript Preparation

During the preparation of this manuscript, we used Perplexity (GPT-5.1, Perplexity AI, 2026) for language editing. All outputs generated by this tool were reviewed and edited by the authors. The authors take full responsibility for the content.

## Abbreviations

AdjLACA: Adjusted laypeople casualty assessment algorithm
KL Divergence: Kullback-Leibler-Divergence
LACA: Laypeople casualty assessment algorithm
mSTaRT: Modified simple triage and rapid treatment
PRIOR: Primäres ranking zur initialen orientierung im rettungsdienst (primary ranking to initial orientation in rescue services)
STaRT: Simple triage and rapid treatment
